# Supplementary material for: Control of bacterial immune signaling by a WYL domain transcription factor
Source: Nucleic Acids Res. 2022 May 10;50(9):5239–50. doi: 10.1093/nar/gkac343 (PMC9122588; doi:10.1093/nar/gkac343)
Supplement: gkac343_Supplemental_Files [file gkac343_supplemental_files.zip › Supplement_May6.pdf]

Supplemental Data

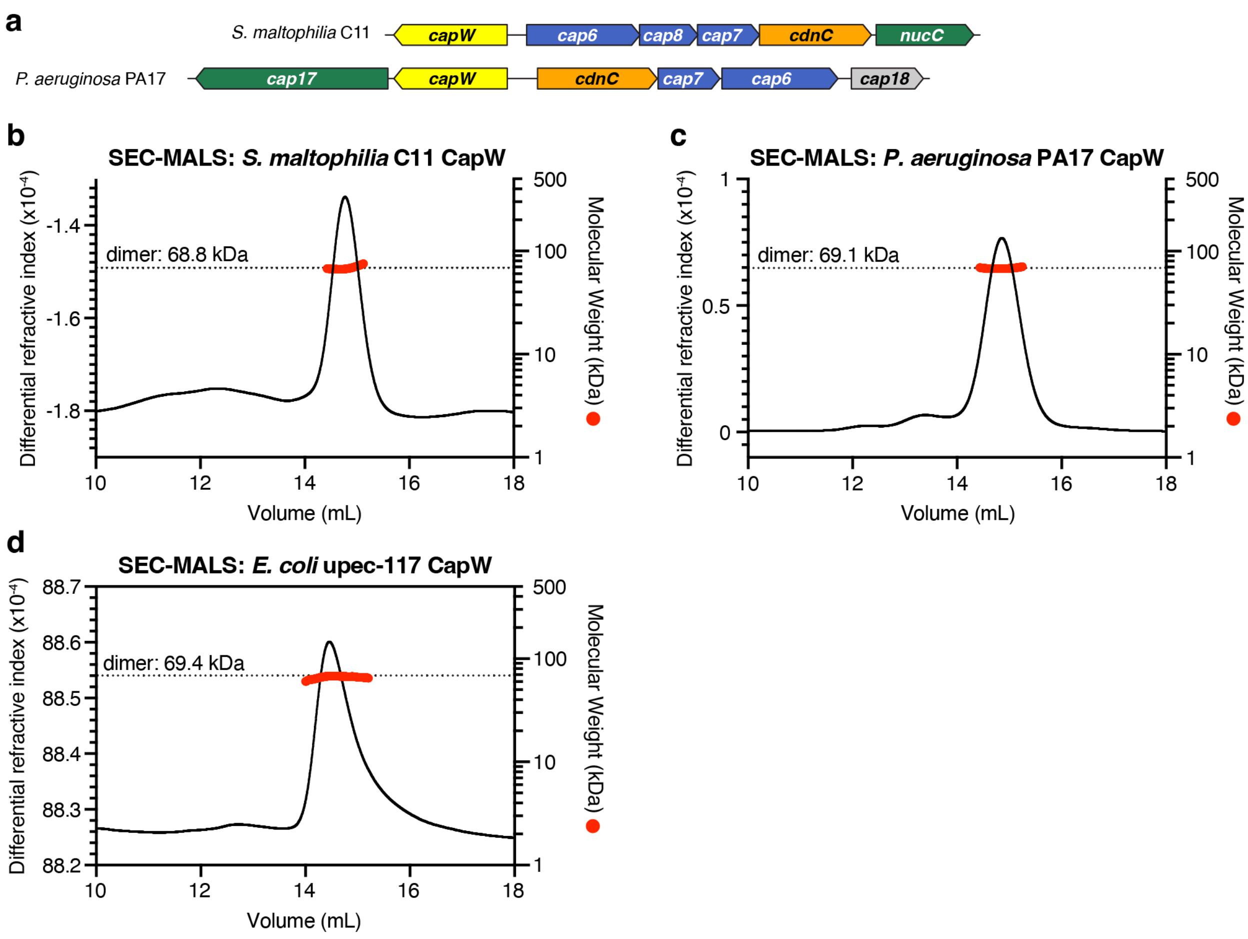

Figure S1. Identification and characterization of CBASS-associated CapW

(a) Operon schematics of CBASS systems from *P. aeruginosa* PA17 and *S. maltophilia* C11, colored as in **Fig. 1a**. (b) Size exclusion chromatography coupled to multi-angle light scattering (SEC-MALS) analysis of purified *Sm* CapW. The measured molecular weight (62.2 kDa; red line) is consistent with a homodimer (68.8 kDa; dotted black line). (c) SEC-MALS analysis of purified *Pa* CapW. The measured molecular weight (68.2 kDa; red line) is consistent with a homodimer (69.1 kDa; dotted black line). (d) SEC-MALS analysis of purified *Ec* CapW. The measured molecular weight (66.1 kDa; red line) is consistent with a homodimer (69.4 kDa; dotted black line).

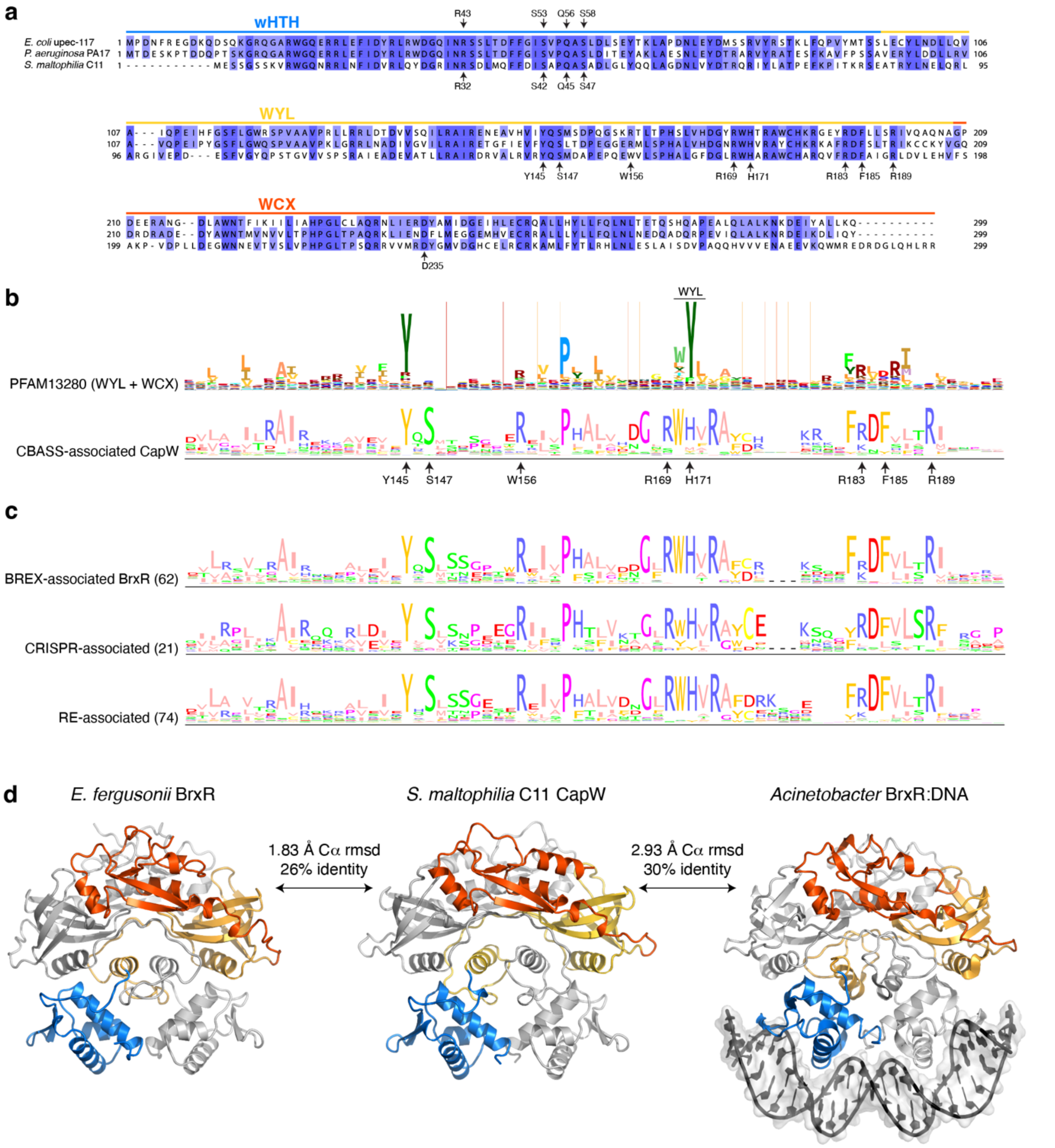

Figure S2. Sequence alignments of CapW and related transcription factors

(a) Sequence alignment of CapW from *E. coli* upec-117, *P. aeruginosa* PA17, and *S. maltophilia* C11. Blue, yellow, and orange lines indicate the extent of the wHTH, WYL, and WCX domains, respectively. wHTH domain residues involved in DNA binding are labeled for *Ec* CapW and *Sm* CapW. Also labeled are putative ligand-binding and allosteric-regulation residues in the WYL and WCX domains of *Sm* CapW. (b) Sequence logos of PFAM13280 (assembled from ~18,000 WYL-WCX domain containing proteins; www.pfam.org) and 160 CBASS-associated CapW proteins (generated with JALVIEW from the proteins listed in **Table S1**) (41). Key putative ligand-binding site residues of *Sm* CapW are labeled. (c) Sequence logos of BrxR (62 sequences) and related WYL domain transcription factors associated with CRISPR/Cas

(21 sequences) and restriction-modification systems (74 sequences), as identified in Picton et al. (14). (d) Comparison of the Sm CapW structure (center) with those of *E. fergusonii* BrxR (PDB ID 7QFZ; (14)) and DNA-bound *Acinetobacter* BrxR (PDB ID 7T8K; (13)). All three proteins are shown in the same orientation, with one protomer gray and the other colored as in **Fig. 3a**. Despite relatively low sequence identity with BrxR, *Sm* CapW shows high overall structural similarity to both structures.

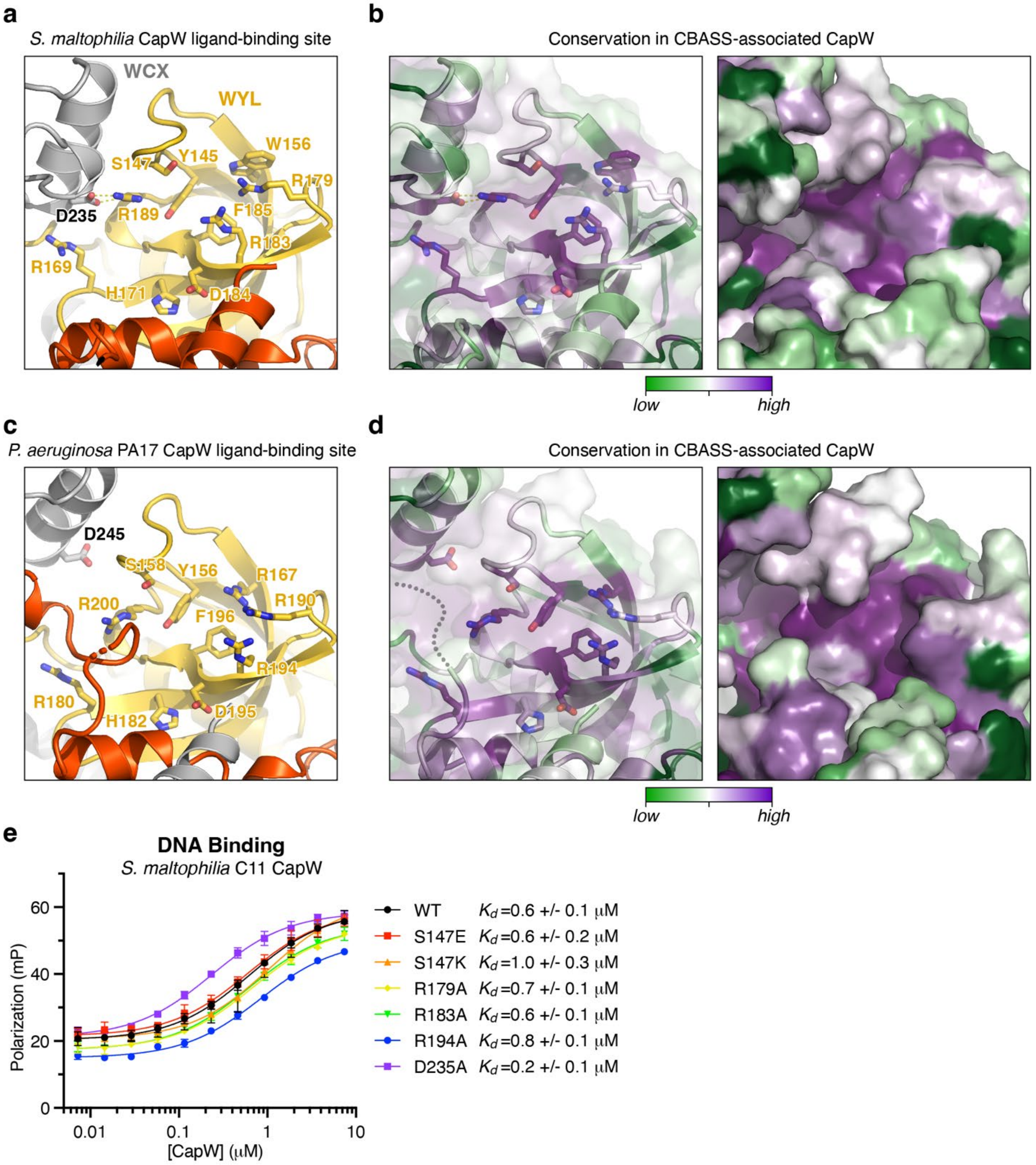

Figure S3. Surface conservation of the CapW putative ligand binding site

(a) Top-down view of the putative ligand-binding site of *Sm* CapW, with WYL domain colored yellow and dimer-related WCX domain gray. (b) View equivalent to panel (a), colored by conservation among 160 CBASS-associated CapW proteins (green: poorly conserved; purple: highly conserved); conservation scores calculated using the ConSurf server (42). (c) Top-down view of the putative ligand-binding site of *Pa* CapW, with WYL domain colored yellow and dimer-related WCX domain gray. (d) View equivalent to

panel (c), colored by conservation among 160 CBASS-associated CapW proteins. For clarity, residues 276-285 have been removed from these panels (gray dotted line). (e) Fluorescence polarization DNA binding assay with *S. maltophilia* C11 CapW (wild-type and indicated mutants of the conserved putative ligand-binding surface) binding DNA Probe #1 (**Fig. 2b**).

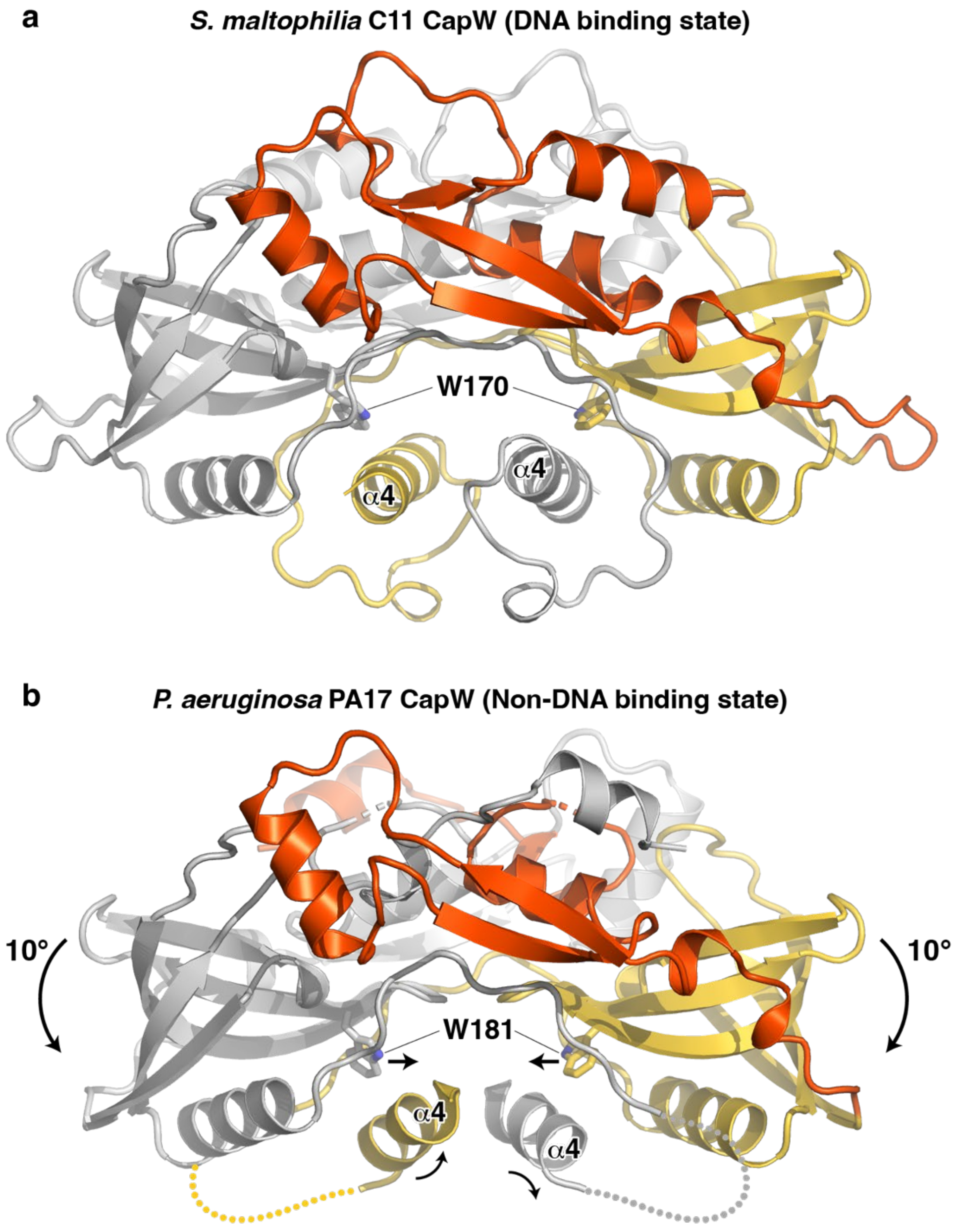

Figure S4. Conformational changes in CapW are driven by WYL domain motions

(a) Front view of the *Sm* CapW dimer, with wHTH domains omitted for clarity. Shown in sticks is residue W170 for both monomers, which packs against the wHTH-WYL linker α4 helix. (b) Front view of the *Pa* CapW dimer, with wHTH domains omitted for clarity. Shown in sticks is residue W181 for both monomers. Compared to the structure of *Sm* CapW, the two W181 residues are closer together, driving rotation of the α4 helix (indicated by arrows) and rotation of the associated wHTH domains (not shown).

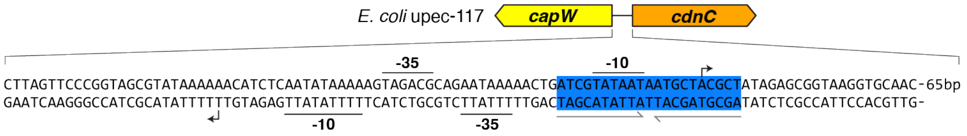

Figure S5. Promoter structure of *E. coli* upec-117 CBASS

Diagram of the *E. coli* upec-117 CBASS promoter showing key sequences (-35 site, -10 site, and transcription start site indicated by arrow) for the top strand (driving core CBASS expression) and the bottom strand (driving *capW* and *cap17* expression). Promoter sequences (-35, -10, and TSS sites) were predicted with the BPROM server (40). Shown in blue is the palindromic sequence that CapW binds.

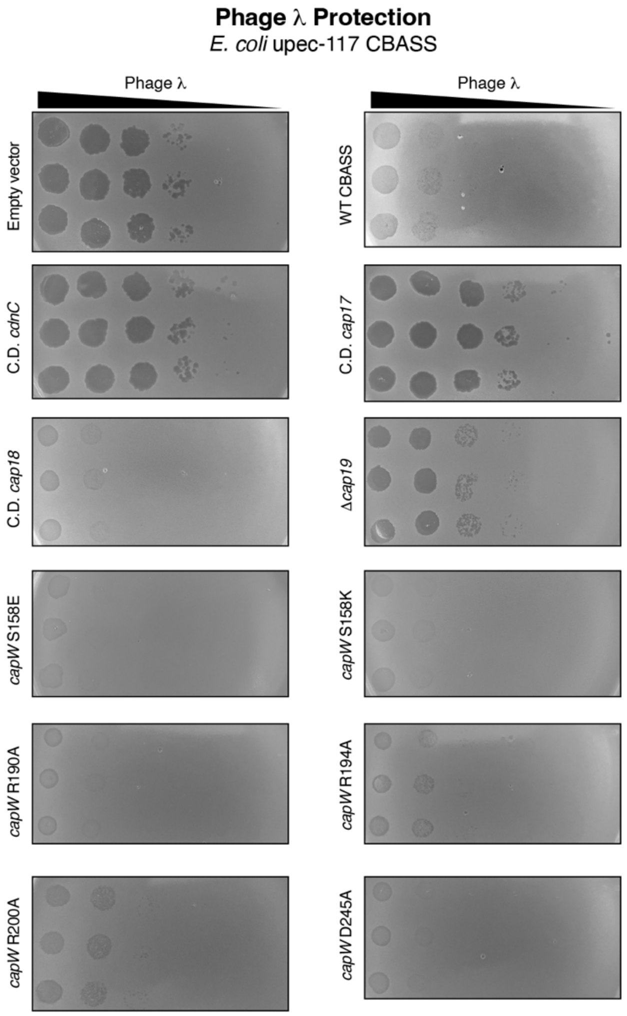

Figure S6. Phage protection by *E. coli* upec-117 CBASS

Plaque assays performed with phage λ cI- on *E. coli* JP313 cells with a vector encoding the *E. coli* upec-117 CBASS system (WT CBASS) or the indicated mutants. Each assay was performed in triplicate with six 10-fold dilutions of phage, then plaques were counted in the highest dilution with visible plaques for each strain. For several strains (C.D. *cap18* and *capW* S158E/S158K/R190A/D245A), plaques uniform clearing was observed for the two most concentrated phage dilutions, but not after. For quantitation (**Fig. 5e**), these were counted as a single plaque in the third dilution.

Supplemental Tables

Table S1. CBASS systems with associated CapW

(see attached Excel sheet)

Table S2. CBASS-associated regulators and effectors

Gene name	Effector/Regulator	Description	Structure(s)	Reference
Cap1/CapV	Effector	Phospholipase	n/a	(38)
Cap2	Regulator (Type II)	Ubiquitin E2/E1-like	n/a	(4,43)
[a] Cap2b	Regulator (Type II - short)	Ubiquitin E2-like	n/a	(4,10)
Cap3	Regulator (Type II)	JAB/JAMM (iso)peptidase-like	n/a	(4,43)
Cap4	Effector	SAVED endonuclease	6WAN, 6VM6, 6VM5, 6WAM	(44)
Cap5	Effector	HNH endonuclease	7RWM, 7RWK	(44,45)
Cap6	Regulator (Type III)	Trip13	6PB3, 6P8V	(7)
Cap7	Regulator (Type III)	HORMA1 (in 1-HORMA systems); HORMA2 (in 2-HORMA systems)	6P8O, 6P8P, 6P8R, 6P8S, 6P8U, 6P80, 6U7B, 6P8V	(7)
Cap8	Regulator (Type III)	HORMA3 (2nd HORMA in 2-HORMA systems)	6P8S	(7)
Cap9	Regulator (Type IV)	QueC	n/a	(10)
Cap10	Regulator (Type IV)	TGT	n/a	(10)
Cap11	Regulator (Type IV)	N-glycosylase/DNA lyase (OGG)	n/a	(10)
Cap12	Effector	TIR-STING	6WT4, 6WT5	(11)
Cap13	Effector	TM-STING	n/a	(11)
Cap14	Effector	TM-SAVED	n/a	(46)
Cap15	Effector	TM-β-barrel	7N34, 7N35	(46)
Cap16	Effector	TM-NUDIX	n/a	(46)
NucC	Effector	endonuclease	6P7O, 6P7Q, 6P7P, 6Q1H, 6UXF, 6UXG	(39)
[a] Cap17	Effector	MTA/SAH nucleoside phosphorylase	n/a	(10), this study
[a] Cap18	*unknown*	3'-5' exonuclease	n/a	(10), this study
[a] Cap19	*unknown*	3-transmembrane protein	n/a	this study

[a] Gene names coined in this study.

Table S3. Crystallographic data collection and refinement

	***Sm* CapW**	***Pa* CapW SeMet**
**Data collection**		
Synchrotron/Beamline	ALS 5.0.2	APS 24ID-E
Date collected	4/25/2021	8/11/2019
Resolution (Å)	94.2 - 1.89	100 - 2.30
Wavelength (Å)	1.00003	0.92918
Space Group	$P6_522$	$P6_122$
Unit Cell Dimensions (a,b,c) Å	112.97, 112.97, 188.39	87.66, 87.66, 199.70
Unit Cell Angles (α,β,γ)°	90, 90, 120	90, 90, 120
I/σ (last shell)	14.1 (1.0)	19.9 (1.6)
[a] R_{sym} (last shell)	0.131 (2.736)	0.111 (2.549)
[b] R_{meas} (last shell)	0.135 (2.837)	0.114 (2.618)
[c] $CC_{1/2}$, last shell	0.999 (0.552)	1.000 (0.897)
Completeness (last shell) %	100.0 (100.0)	99.9 (98.9)
Number of reflections	1051782	402418
unique	57481	21112
Multiplicity (last shell)	18.3 (14.3)	19.1 (19.1)
**Refinement**		
Resolution (Å)	56.5 - 1.89	60 - 2.30
No. of reflections	57356	20964
working	54434	19086
free	2922	1878
[d] R_{work} (%)	19.65 (32.55)	22.29 (34.28)
[d] R_{free} (%)	21.44 (28.53)	24.81 (35.05)
**Structure/Stereochemistry**		
Number of atoms	4821	4265
hydrogen	2269	2104
solvent	232	15
r.m.s.d. Bond lengths (Å)	0.0069	0.0062
r.m.s.d. Bond angles (°)	0.85	0.77
Rotamer outliers (%)	1.22%	1.36%
Ramachandran (%)		
Favored	98.23%	98.85%
Allowed	1.77%	1.15%
Outliers	0.00%	0.00%
MolProbity score	0.88	1.36
MolProbity Clashscore	1.09	4.94
PDB ID	7TB6	7TB5
SBGrid Data Bank ID	870	869

[a] $R_{sym} = \sum\sum_j |I_j - \langle I \rangle| / \sum I_j$, where I_j is the intensity measurement for reflection j and $\langle I \rangle$ is the mean intensity for multiply recorded reflections.

[b] $R_{meas} = \sum_h [\sqrt{(n/(n-1))} \sum_j [I_{hj} - \langle I_h \rangle] / \sum_{hj} \langle I_h \rangle$, where I_{hj} is a single intensity measurement for reflection h, $\langle I_h \rangle$ is the average intensity measurement for multiply recorded reflections, and n is the number of observations of reflection h.

[c] $CC_{1/2}$ is the Pearson correlation coefficient between the average measured intensities of two randomly-assigned half-sets of the measurements of each unique reflection (47).

[d] $R_{work, free} = \sum||F_{obs}| - |F_{calc}||/|F_{obs}|$, where the working and free R-factors are calculated using the working and free reflection sets, respectively.
[f] Coordinates and structure factors have been deposited in the RCSB Protein Data Bank (http://www.rcsb.org).
[g] Diffraction data have been deposited with the SBGrid Data Bank (http://data.sbgrid.org) with the noted accession codes.

Supplemental References

41. Waterhouse, A.M., Procter, J.B., Martin, D.M.A., Clamp, M. and Barton, G.J. (2009) Jalview Version 2--a multiple sequence alignment editor and analysis workbench. *Bioinformatics*, **25**, 1189-1191.
42. Ashkenazy, H., Erez, E., Martz, E., Pupko, T. and Ben-Tal, N. (2010) ConSurf 2010: calculating evolutionary conservation in sequence and structure of proteins and nucleic acids. *Nucleic Acids Res*, **38**, W529--533.
43. Iyer, L.M., Burroughs, A.M. and Aravind, L. (2006) The prokaryotic antecedents of the ubiquitin-signaling system and the early evolution of ubiquitin-like β-grasp domains. *Genome Biol*, **7**, R60.
44. Lowey, B., Whiteley, A.T., Keszei, A.F.A., Morehouse, B.R., Mathews, I.T., Antine, S.P., Cabrera, V.J., Kashin, D., Niemann, P., Jain, M. et al. (2020) CBASS Immunity Uses CARF-Related Effectors to Sense 3'-5'- and 2'-5'-Linked Cyclic Oligonucleotide Signals and Protect Bacteria from Phage Infection. *Cell*, **182**, 38-49 e17.
45. Fatma, S., Chakravarti, A., Zeng, X. and Huang, R.H. (2021) Molecular mechanisms of the CdnG-Cap5 antiphage defense system employing 3',2'-cGAMP as the second messenger. *Nat Commun*, **12**, 6381.
46. Duncan-Lowey, B., McNamara-Bordewick, N.K., Tal, N., Sorek, R. and Kranzusch, P.J. (2021) Effector-mediated membrane disruption controls cell death in CBASS antiphage defense. *Mol Cell*, **81**, 5039-5051.
47. Karplus, P.A. and Diederichs, K. (2012) Linking crystallographic model and data quality. *Science*, **336**, 1030-1033.
